# The influence of teacher support on student engagement in physical education among college students: The mediating effects of autonomous motivation and self-efficacy

**DOI:** 10.1371/journal.pone.0331876

**Published:** 2025-09-18

**Authors:** Qi Guo, Xiaopeng Wang, Zhendong Gao, Jianxin Gao, Xiaofei Lin, Shamsulariffin Samsudin

**Affiliations:** 1 Department of Sports Studies, Faculty of Educational Studies, Universiti Putra Malaysia, Serdang, Selangor, Malaysia; 2 College of Physical Culture, Jiangxi Teachers College, Yingtan, Jiangxi, China; University of Tartu, ESTONIA

## Abstract

Student engagement is a malleable behavior shaped by instructional environments and widely regarded as a key component of higher education quality. Nevertheless, many college students have inadequate positive experiences with physical education classes, characterized by a lack of engagement. Therefore, framed within the self-determination theory and social cognitive theory, the present empirical research investigates how autonomy, relatedness, and competence support are associated with student engagement during physical education learning, focusing on the mediating variables of autonomous motivation and self-efficacy. Data collection involved a cross-sectional survey administered to undergraduates from four colleges located in Jiangxi Province, China. After excluding invalid questionnaires, 406 eligible responses were included in the data analysis. Structural equation modelling was then constructed through AMOS 26.0 for hypothesis testing. According to the findings, teacher autonomy and relatedness support were significantly related to student engagement, while competence support showed no significant correlation. Additionally, the pathways from autonomy and competence support to student engagement were both mediated by autonomous motivation. However, its mediating role was not supported in the link between relatedness support and student engagement. Furthermore, teacher autonomy, competence, and relatedness support all significantly influence student engagement via self-efficacy. The present study explores the relational mechanisms linking teacher support to student engagement in college physical education. These results contribute to refining teaching strategies to enhance student engagement while providing insights into optimally motivating instruction in higher education physical education teaching.

## 1 Introduction

Student engagement has been widely regarded as vital to achieving academic success [1]. Increasingly, research has revealed that enhancing student engagement leads to diverse educational benefits, from academic achievement [2] and persistence [3] to future success in life [4]. Collectively, this line of evidence underscores the pivotal role of student engagement through all stages of the learning process. By contrast, students with low engagement typically have lower academic expectations. They tend to experience negative emotional states, including anxiety and boredom, which may result in disruptive behaviors and even dropping out of school prematurely [5,6]. Encouraging active student engagement is a primary aim of Physical Education (PE), as students need to participate to some extent in school PE in order to achieve the ability to maintain healthy exercise. Highly engaged students in PE classes demonstrate diligence in acquiring knowledge and skills, and acknowledge the significance of maintaining lifelong physical activity to promote physical and mental well-being [7]. Hence, fostering student engagement in PE programs warrants substantial attention from educational practitioners.

In the landscape of contemporary higher education, student engagement has gained widespread attention as a critical measure of educational quality [8]. However, the increasing scale and complexity of higher education institutions gradually lead to the differentiated development of student engagement [9]. Many college students in PE classes exhibit inattentiveness and a lack of effort toward learning tasks [10]. In China, due to shortcomings in the college entrance examination evaluation system, PE holds a marginal position within the broader education system. Consequently, a considerable number of students enter college with low levels of engagement in PE [11]. They often exhibit a passive and utilitarian attitude in PE classes, participating primarily to earn course credits [12]. On the other hand, Low levels of engagement in physical education classes serve as a precursor to students’ health issues and behavioral problems [5]. More broadly, sustained low engagement of college students is associated with crime, substance abuse, and depression [13,14]. Simply increasing the number of PE courses or introducing different instructional equipment may not effectively enhance student engagement [15]. Therefore, investigating predictors of student engagement during PE sessions is critical for enhancing the educational value of PE programs at the collegiate level.

Student engagement is a malleable behavior, which means that teachers can make appropriate adjustments at the instructional level to stimulate student engagement [16]. As posited by Self-Determination Theory (SDT), teachers supporting students’ basic psychological needs, which consist of autonomy, competence, and relatedness, can facilitate internalizing external motivation, thereby promoting student engagement [17]. In contrast, if students’ psychological needs are obstructed, these thwarted needs potentially initiate a decline in intrinsic motivation, resulting in negative behaviors such as low engagement, fear of failure, and avoidance of challenges [18]. Prior studies suggest that teacher support significantly predicts student engagement [19], including in PE settings [20,21]. Thus, creating a supportive teaching environment is essential for fostering student engagement. Nevertheless, the current content of PE in higher education tends to exhibit formalism, with instructors often neglecting students’ intrinsic psychological needs and emotional interactions [22]. Although this topic is gaining increasing attention, empirical studies focusing on PE contexts remain limited compared to traditional classroom settings and lack consideration of all dimensions of teacher support [23]. Compared to research conducted at the middle and high school levels, studies examining students in higher education have emerged relatively late, and empirical evidence remains limited [1]. Given this context, this research draws on SDT to comprehensively explore the mechanisms linking teacher support (autonomy, competence, and relatedness) to student engagement in college PE classes.

From the perspective of Social Cognitive Theory (SCT), self-efficacy functions as a key personal factor in predicting behavioral outcomes, while also being shaped by environmental influences [24]. Li et al. [25] indicate that teacher support for students’ psychological needs, including collaborative problem-solving discussions and valuing student perspectives, contributes to the strengthening of students’ self-efficacy. Students characterized by higher self-efficacy are inclined to maintain active engagement in learning tasks [26,27], whereas those with lower self-efficacy levels often exhibit apathy [28]. Unlike other disciplines, students’ self-efficacy in PE is more outwardly visible and less easily hidden because physical performance is accompanied by immediate, observable feedback. Nevertheless, the relevance of self-efficacy in shaping teacher support’s connection to student engagement in PE has yet to be thoroughly examined. Autonomous motivation facilitates a range of behavioral and psychological processes by reflecting individuals’ initiative and sense of control over their actions [29]. Empirical studies demonstrate that students exhibiting strong autonomous motivation can internalize external incentives, developing intrinsic drives that sustain optimal engagement [30,31]. It is worth noting that Chinese education tends to emphasize collectivism, and students’ learning motivation is usually driven by external factors [32]. Therefore, the stimulation of students’ autonomous motivation to improve their engagement in PE within a collectivist cultural context warrants further investigation.

**Fig 1:**
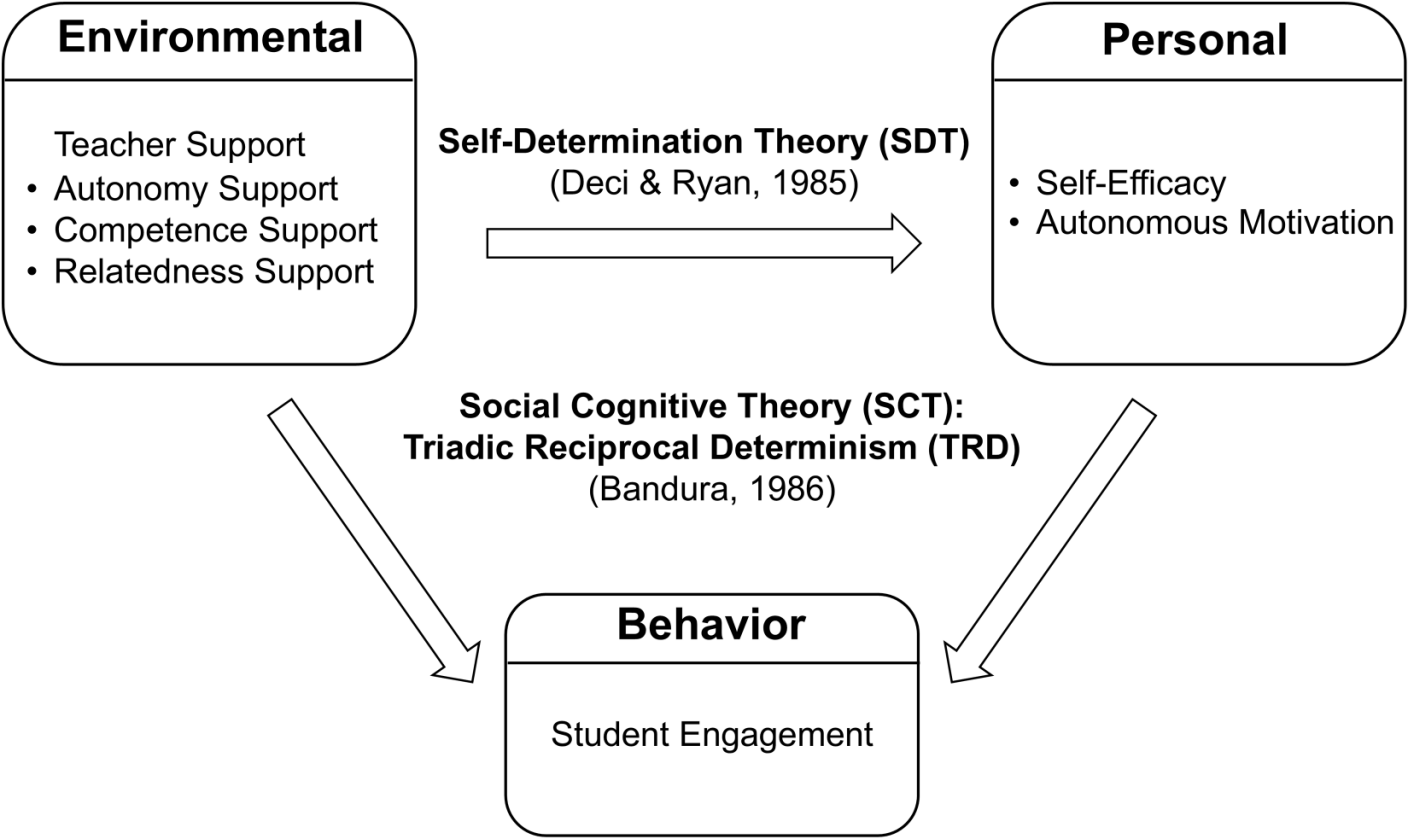
Theoretical framework of current study.

SCT and SDT have been widely applied in educational contexts to explain learning motivation process [33]. SDT primarily emphasizes that the satisfaction of three basic psychological needs, namely autonomy, competence, and relatedness, serves as the foundation for the internalization of motivation. By satisfying these needs, students are more likely to transform external tasks or behavioral demands into autonomous motivation rooted in personal values, thereby demonstrating sustained learning behaviors [34]. In contrast, SCT highlights self-efficacy, generally interpreted as one’s perceived capability to accomplish specific tasks, and identifies it as a key determinant of initiating learning behaviors [33]. Put differently, autonomous motivation reflects an internalized willingness to act (“I want”), whereas self-efficacy represents a subjective belief in one’s ability to succeed (“I can”). These two constructs correspond to the core theoretical features of motivational quality and efficacy beliefs, respectively, and together offer a complementary perspective for understanding the psychological mechanisms underlying student engagement in physical education. In addition, Triadic Reciprocal Determinism (TRD), a core principle of SCT, conceptualizes behavior, personal factors, and environmental influences as dynamically interconnected elements. Within this triadic system, self-efficacy is categorized as a personal factor and functions as a mediator linking environmental input to behavioral outcomes [35]. Accordingly, this study adopts TRD as the basis for its theoretical framework (Fig 1), in which teacher support represents the environmental component, self-efficacy and autonomous motivation constitute the personal component, and student engagement reflects the behavior component.

Drawing on SDT and SCT, this study focuses on investigating how teacher support relates to student engagement in PE, with autonomous motivation and self-efficacy as mediators. The current study helps reveal the mechanisms that influence student engagement, providing a theoretical basis for addressing the negative state of college students in PE classes and serving as a reference for promoting high-quality PE instruction.

## 2 Literature review

### Teacher support and student engagement

Student engagement entails the quality of a student’s connection or involvement with people, places, goals, values, and tasks throughout the educational process [36]. It is a multifaceted construct typically divided into three components: behavioral, emotional, and cognitive. These aspects reflect students’ behavioral performance, emotional responses, and use of self-regulation strategies in academic settings [16]. Among the external factors affecting student engagement, the importance of teacher support cannot be overlooked. The concept of teacher support is derived from Self-Determination Theory (SDT), which underscores the existence of three basic psychological needs in every individual: autonomy, competence, and relatedness [37]. Teachers can activate students’ intrinsic motivational resources by supporting their basic psychological needs, thereby sustaining their ongoing engagement [38]. Throughout this process, teachers are not only technical instructors but also creators of a supportive learning environment. By fostering a positive interpersonal interactions, they enhance students’ emotional experiences, creating conditions that encourage the development of positive learning behaviors [39]. Specifically, autonomy support encompasses a range of teacher behaviors aimed at fostering students’ sense of volition and personal agency in the learning process. It refers to teachers providing students with space for self-directed decision-making, recognizing students’ perspectives, employing instructional strategies that align with their interests, and clearly communicating the value and purpose of learning tasks [40]. Competence support involves teachers assigning appropriately challenging tasks, providing directive feedback, and acknowledging students’ efforts to enhance their perceived effectiveness in relevant learning tasks [40]. Relatedness support is reflected in educators fostering positive teacher–student relationships through emotionally connective behaviors, including conveying warmth and care, providing unconditional positive regard, and establishing reciprocal interaction grounded in respect and empathy [40].

Current research generally recognizes that teacher support plays an integral role in improving student engagement. A study in mathematics classrooms suggested that student engagement improved when teachers responded to learners’ psychological needs [41]. Chiu [42] revealed that, even without direct interaction, teacher support significantly promoted student engagement in remote education during the COVID-19 outbreak. According to Patall et al. [43], teachers’ autonomy support shows a strong association to students’ intrinsic motivation and engagement. Likewise, teacher competence support improves engagement and academic performance [44]. Additionally, teacher relatedness support increases students’ motivation and engagement [45]. Research on related topics is increasingly focused on the field of PE. Otundo and Garn [20] found that a need-supportive environment created by teachers fosters a favorable climate for teacher-student relationships, significantly enhancing middle school students’ interest in and engagement with PE. Leo et al. [36] investigated 2065 adolescent students and analyzed need-supportive and need-thwarting instruction. The analysis indicated that students in the high-low group (high need-supportive and low need-thwarting instruction) had higher behavioral and emotional engagement levels in PE. Exploration of specific aspects of teacher support in PE has occurred in multiple studies. In an experimental study using a PE teaching-style video for intervention, secondary school students who watched autonomy-supportive teaching clips reported higher engagement levels than those who watched controlling teaching clips [46]. Yoo [47] suggested that autonomy support from PE teachers can increase student engagement, particularly for students with positive emotions in PE. In addition, relatedness support significantly predicted emotional and behavioral engagement among female high school students in PE. Conversely, students lacking relatedness support were likelier to lack enthusiasm and detachment from PE classes [48]. Current research pays relatively insufficient attention to teacher competence support, and its specific role in PE instruction remains unclear, which may be due to the implementation differences of this support strategy in various teaching scenarios.

However, some studies have also reported negative results. González-Peño [49] reported that PE teachers’ autonomy support did not relate to students’ behavioral engagement. The literature explains that students may view the autonomy provided by teachers as disorganized, which may lead to students showing waiting behavioral traits. Similarly, a prior investigation involving obese Chinese adolescents indicated that the support of PE teachers does not directly affect students’ engagement [50]. Considering the different interpretations regarding how teacher support influences student engagement in PE provided by existing literature, further research could help clarify the underlying mechanisms. Accordingly, the following hypothesis is formulated:

*H1: Autonomy support (H1a), competence support (H1b), and relatedness support (H1c) positively predict student engagement.*

### Mediating role of autonomous motivation

Autonomous motivation embodies a highly self-determined form of motivation developed by interacting with an individual’s internal psychological and external environmental factors [51]. Autonomous motivation denotes one’s inclination to voluntarily engage in a task out of their own will and choice [52]. It is the driving factor for individuals to maintain specific behaviors and comprises three dimensions: identified regulation, integrated regulation, and intrinsic motivation [53]. As outlined in SDT [54], identified regulation is characterized by individuals recognizing the importance of their behavior. Integrated regulation involves aligning behavior with one’s values. Intrinsic motivation refers to people spontaneously engaging in behavior following their innate interests and satisfactions.

It is well-documented that teacher support positively influences the formation of students’ autonomous motivation. Autonomy-supportive teaching positively predicts students’ extracurricular physical activity motivation [55]. PE teachers’ relatedness support allows students to experience caring and personalized attention, which leads to stronger autonomous motivation in physical activities [56]. Moreover, empirical literature suggests that students with autonomous motivation exhibit significantly increased engagement in learning. To illustrate, a study by Ganotice et al. [57] on interdisciplinary learning demonstrated that students with autonomous motivation tended to stay focused, which benefited their emotional engagement. In a study involving 1,120 students, Leo et al. [58] revealed that students exhibiting strong autonomous motivation were more resilient to frustration, thereby demonstrating more engagement. An investigation in an online EFL setting reveals that students’ perception of teacher support was positively associated with learning engagement, with this connection significantly mediated by autonomous motivation [59]. Polet et al. [60] reported that teacher support serves as a positive contributor to the development of students’ autonomous motivation, indirectly shaping their intention to participate in physical activities. Even for less appealing tasks, teacher support for psychological needs can stimulate students’ autonomous motivation, which in turn can increase their level of engagement [61]. SDT posits that the satisfaction of basic psychological needs serves as a precursor to the internalization of motivation [34]. Accordingly, physical education teachers can enhance students’ autonomous motivation by engaging in need-supportive behaviors, thereby promoting more positive learning outcomes [62]. Chinese college students are situated within a collectivist cultural context, where group-oriented values may lessen the salience of individual autonomy [63]. However, multiple meta-analyses have demonstrated that the beneficial effects of psychological need support in educational settings are applicable across cultural contexts [64–66]. Based on the above, autonomous motivation may serve as a mediator linking teacher support (autonomy, competence, and relatedness support) to student engagement in PE among college students. Accordingly, we hypothesize the following:

*H2: Autonomy support (H2a), competence support (H2b), and relatedness support (H2c) have mediation effects through autonomous motivation on student engagement.*

### Mediating role of self-efficacy

As a key psychological construct in learning, self-efficacy denotes an individual’s perceived ability and skill performance in learning [67]. Over recent decades, it has emerged as a vital influence on university-level academic performance [68]. Bandura [69] emphasized in his Social Cognitive Theory (SCT) that the learning process can be explained through the interactions between the environment, person, and behavior. Positioned within Bandura’s triadic framework, self-efficacy is a pivotal personal construct that is both influenced by environmental conditions and affects the learner’s behavior [70]. Within educational environments, teacher support greatly contributes to students’ awareness of their competencies and the development of self-efficacy. Specifically, teachers may establish a supportive learning atmosphere by setting appropriate learning goals and providing individualized instruction. This support enhances students’ self-perceptions and advances their self-efficacy during the course [71]. Recent research indicates that the freedom of communication and choice embodied in autonomy-supportive teaching helps promote self-efficacy as students develop a clearer awareness of their competence on a subjective level [72]. Students with elevated self-efficacy beliefs tend to perceive academic challenges not as threats but as opportunities to grow, which in turn fosters adaptive coping mechanisms and a greater willingness to exert effort in overcoming learning obstacles [73]. This psychological trait not only enhances students’ confidence in accomplishing a specific learning task but also further promotes their persistence and engagement [74]. When teachers offer competence support through consistent guidance and structured feedback that reinforces students’ sense of capability, learners typically build strong self-efficacy, thereby promoting deeper engagement in learning activities [75]. Through network analysis, Hu et al. [71] identified self-efficacy as an intermediary in the link between teacher autonomy support and learning engagement. In light of the above, there is a research gap in the indirect pathway by which teacher support affects student engagement via self-efficacy in PE settings. Therefore, the subsequent hypotheses are posited:

*H3: Autonomy support (H3a), competence support (H3b), and relatedness support (H3c) have mediation effects through self-efficacy on student engagement.*

**Fig 2:**
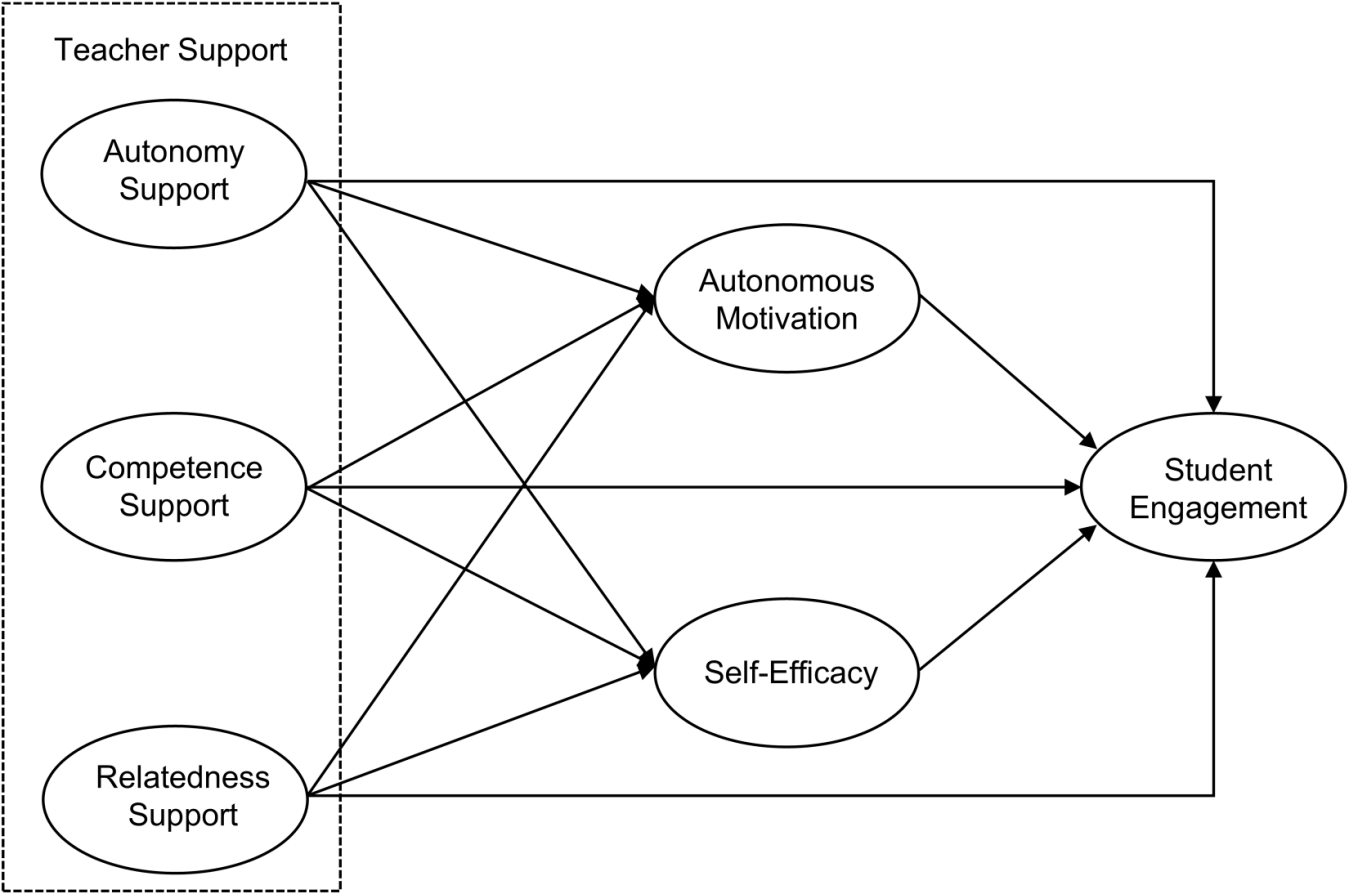
Conceptual model of current study.

In summary, while the impact of teacher support on shaping student engagement has received considerable global attention, the potential mechanisms in the context of PE remain inadequately explored. To this end, Fig 2 illustrates the conceptual model developed to examine how teacher support (autonomy, competence, and relatedness) influences student engagement through the mediation effects of autonomous motivation and self-efficacy.

## 3 Method

### Participants

The research employed stratified random sampling to conduct a questionnaire survey on first-year to third-year undergraduate students enrolled at four comprehensive colleges in Jiangxi Province, China, from March 11–22, 2024. We focused exclusively on undergraduate students, given that most Chinese colleges do not offer compulsory PE courses for the master’s and doctoral levels [76]. Following a thorough ethical review, the Universiti Putra Malaysia Ethics Committee approved this research (JKEUPM-2023–685), which was undertaken in compliance with the Declaration of Helsinki. Prior to commencing the survey, participants were assured that their responses would be kept confidential and used exclusively for academic research purposes. Furthermore, to help participants clearly grasp the study objectives and procedures, we provided a detailed informed consent form outlining rights and privacy, designed to uphold the right to information and emphasize voluntary participation. Written informed consent was obtained from all participants, and the questionnaire was completed anonymously. This rigorous process is designed to guarantee that our research adheres to ethical principles and regulations and to protect the rights of the subjects participating in the study.

The survey questionnaires were distributed on-site, and 450 college students completed the survey. Participants came from different faculties that did not major in PE. After excluding 44 invalid questionnaires, the researchers collected 406 with a valid response rate of 90.2%. The main criteria for excluding invalid questionnaires were: (1) the completion time was too short; (2) there was excessive repetition of the same answers or patterns; (3) there were unanswered items. The participants comprised 191 female (47.0%) and 215 male students (53.0%), aged 17–22 years. By academic year, 159 (39.2%) were first-year, 130 (32.0%) second-year, and 117 (28.8%) third-year undergraduates.

### Instruments

All measures used in the study were well-established scales developed by scholars. Since the participants were native Chinese speakers, following Brislin [77], the original scales were translated into Chinese, with a subsequent back-translation into English to confirm semantic consistency. Two bilingual professionals carried out this procedure and resolved discrepancies between the two versions through discussion. Subsequently, a panel of three PE experts reviewed and confirmed the final Chinese version of the questionnaire. Demographic information on respondents’ gender, academic year, was gathered in the initial part of the questionnaire. The second part utilized scales from previous studies to assess teacher support, self-efficacy, autonomous motivation, and student engagement.

#### Teacher support scale.

College students’ views on teacher support were assessed through the instrument proposed by Standage et al. [78]. The measure consisted of three distinct aspects: autonomy support, competence support, and relatedness support. Participants evaluated 24 items assessing this construct through a 5-point Likert response format, anchored at 1 = completely disagree and 5 = completely agree. This scale was widely applied in PE class scenarios [79]. The reliability test yielded a Cronbach’s α coefficient of 0.89, which signifies high internal consistency.

#### Student engagement scale.

The Engagement versus Disaffection with Learning Student Report (EVDLS) and the Metacognitive Strategies Questionnaire were employed to measure student engagement among undergraduates [80,81]. The 10 items in the EVDLS assess students’ behavioral and emotional engagement. Eight items adapted from the Metacognitive Strategies Questionnaire assessed cognitive engagement. Participants evaluated a total of 18 items assessing this construct through a 5-point Likert response format, anchored at 1 = completely disagree and 5 = completely agree. The student engagement scale has been shown to demonstrate good reliability [43]. The reliability test yielded a Cronbach’s α coefficient of 0.84, suggesting high internal consistency.

#### Autonomous motivation scale.

The Behavioural Regulation in Exercise Questionnaire-3 (BREQ-3) evaluated college students’ autonomous motivation [82]. It encompasses three key dimensions: identified regulation, integrated regulation, and intrinsic motivation. Each dimension contained four items. Participants evaluated a total of 12 items assessing this construct through a 5-point Likert response format, anchored at 1 = completely disagree and 5 = completely agree. The BREQ-3 was verified to have satisfactory reliability and validity within a Chinese undergraduate sample [83]. The reliability test yielded a Cronbach’s α coefficient of 0.90, suggesting strong internal consistency.

#### Self-efficacy scale.

College students’ self-efficacy was assessed using the General Self-Efficacy Scale (GSES), created by Schwarzer and Jerusalem [84]. Participants evaluated 10 items assessing this construct through a 5-point Likert response format, anchored at 1 = completely disagree and 5 = completely agree. GSES was identified as highly reliable and valid for the Chinese college student sample [85]. The reliability test yielded a Cronbach’s α coefficient of 0.92, suggesting strong internal consistency.

#### Data analysis.

Statistical analyses were performed with SPSS and AMOS software (both version 26.0). The initial stage involved descriptive and correlational analyses performed in SPSS 26.0. Subsequently, Amos 26.0 was employed to conduct confirmatory factor analysis (CFA) and structural equation modelling. Following the guidance of previous studies [86], items grouped under the same dimension in the multidimensional autonomous motivation and student engagement scales were packaged separately using the internal consistency method. Evaluation of model fit was conducted using six indices: χ2/df, SRMR, RMSEA, CFI, TLI, and IFI. Finally, the mediation analysis used the bias-corrected percentile bootstrap method with 5000 resamples to calculate a 95% confidence interval (CI). If the CI did not contain zero, it indicated a significant mediation effect [87].

## 4 Results

### Common method bias analysis

In light of the exclusive use of self-report measures, this study assessed the presence of common method bias. Based on Harman’s single-factor test, six factors with eigenvalues greater than 1 were extracted, and the variance explained by the dominant factor was 36.81%, falling short of the 40% threshold [88]. Moreover, we employed the unmeasured latent method construct (ULMC) technique [88] to examine common method bias. A seven-factor model was specified on the basis of the original six-factor measurement model by adding a common method factor with all items as indicators. The results indicated that incorporating the common method factor showed no significant improvement in model fit [89] (ΔRMSEA = 0.005, ΔSRMR = 0.003, both < 0.05; ΔCFI = 0.013, ΔTLI = 0.014, both < 0.10). Drawing on the above evidence, the present study did not detect any substantial common method bias.

Additionally, multicollinearity among predictors was tested using the Variance Inflation Factor (VIF), and the results yielded that the VIF value ranged from 1.235 to 2.218, which was smaller than the upper limit of 3.3 recommended by Kock and Lynn [90], indicating no substantial multicollinearity concerns.

### Measurement model

This study validated the measurement model’s composite reliability, convergent validity, and discriminant validity by confirmatory factor analysis (CFA). Five items (AS8, AS11, AS12, AS15, GSE4) were deleted from the first round of CFA due to standardized factor loadings less than the minimum standard of 0.5. The fitting indices demonstrated an acceptable fit of the final measurement model: χ2/df = 2.077, SRMR = 0.0578, RMSEA = 0.052, CFI = 0.928, IFI = 0.928, TLI = 0.921. As shown in Table 1, the CR values of all constructs in this study ranged from 0.804 to 0.928, above the standard value of 0.7, implying that the measurement model employed here is highly reliable. The standardised factor loadings for all items fell between 0.597 and 0.883, and the AVE values for each variable fell between 0.518 and 0.605, exceeding the 0.5 threshold and confirming adequate convergent validity [91]. Discriminant validity was analysed using Fornell and Larcker [92] guidelines and Heterotrait-Monotrait (HTMT) ratios [93]. As shown in Table 2, the AVE square roots exceeded the corresponding correlation coefficients, and all HTMT values were under the 0.9 cutoff value, demonstrating satisfactory discriminant validity.

**Table 1:** Results of measurement model.

Latent Constructs	Observed Variable/Items	FL	CR	AVE
Autonomy Support	AS1	0.856	0.922	0.518
	AS2	0.704		
	AS3	0.706		
	AS4	0.717		
	AS5	0.785		
	AS6	0.631		
	AS7	0.724		
	AS9	0.735		
	AS10	0.669		
	AS13	0.704		
	AS14	0.661		
Competence Support	CS1	0.762	0.850	0.587
	CS2	0.705		
	CS3	0.806		
	CS4	0.788		
Relatedness Support	RS1	0.726	0.872	0.578
	RS2	0.840		
	RS3	0.730		
	RS4	0.705		
	RS5	0.791		
Self-Efficacy	GSE1	0.768	0.928	0.589
	GSE2	0.746		
	GSE3	0.798		
	GSE5	0.707		
	GSE6	0.761		
	GSE7	0.782		
	GSE8	0.702		
	GSE9	0.790		
	GSE10	0.843		
Autonomous Motivation	IDR	0.597	0.818	0.605
	INR	0.883		
	IM	0.824		
Student Engagement	BE	0.624	0.804	0.582
	EE	0.863		
	CE	0.782		

**Table 2:** Discriminant validity.

	AS	CS	RS	GSE	AM	SE
AS	**0.720**	0.415	0.451	0.638	0.432	0.619
CS	0.413	**0.766**	0.363	0.559	0.333	0.404
RS	0.450	0.361	**0.760**	0.606	0.311	0.506
GSE	0.634	0.554	0.606	**0.767**	0.454	0.687
AM	0.402	0.299	0.293	0.413	**0.778**	0.491
SE	0.552	0.348	0.459	0.584	0.389	**0.763**

The diagonal entries are the square root of AVE; The values below the diagonal are the correlation coefficients, and the values above the diagonal are the HTMT ratios; AS, Autonomy Support; CS, Competence Support; RS, Relatedness Support; GSE, General Self-Efficacy; AM, Autonomous Motivation; SE, Student Engagement.

### Descriptive and correlational analyses

Table 3 lists each research variable’s mean, standard deviation, and correlation matrix. Among college students, the mean scores for autonomy support, competence support, relatedness support, self-efficacy, autonomous motivation, and student engagement were 3.35 (±1.05), 3.28 (±1.22), 3.36 (±1.15), 3.32 (±1.13), 3.28 (±0.96), and 3.49 (±0.68), respectively. Notably, all six variables exhibited significant positive correlations. This result suggested that it was appropriate to include these variables when considering the effects pathway of teacher support leading to student engagement.

**Table 3:** Descriptive statistics and correlations matrix.

Variables	M	SD	1	2	3	4	5	6
1. AS	3.35	1.05	**1**					
2. CS	3.28	1.22	0.368**	**1**				
3. RS	3.36	1.15	0.404**	0.313**	**1**			
4. GSE	3.32	1.13	0.590**	0.497**	0.544**	**1**		
5. AM	3.28	0.96	0.372**	0.275**	0.261**	0.391**	**1**	
6. SE	3.49	0.68	0.510**	0.326**	0.405**	0.577**	0.372**	**1**

**p < 0.01; AS, Autonomy Support; CS, Competence Support; RS, Relatedness Support; GSE, General Self-Efficacy; AM, Autonomous Motivation; SE, Student Engagement.

### Hypothesis testing

**Fig 3:**
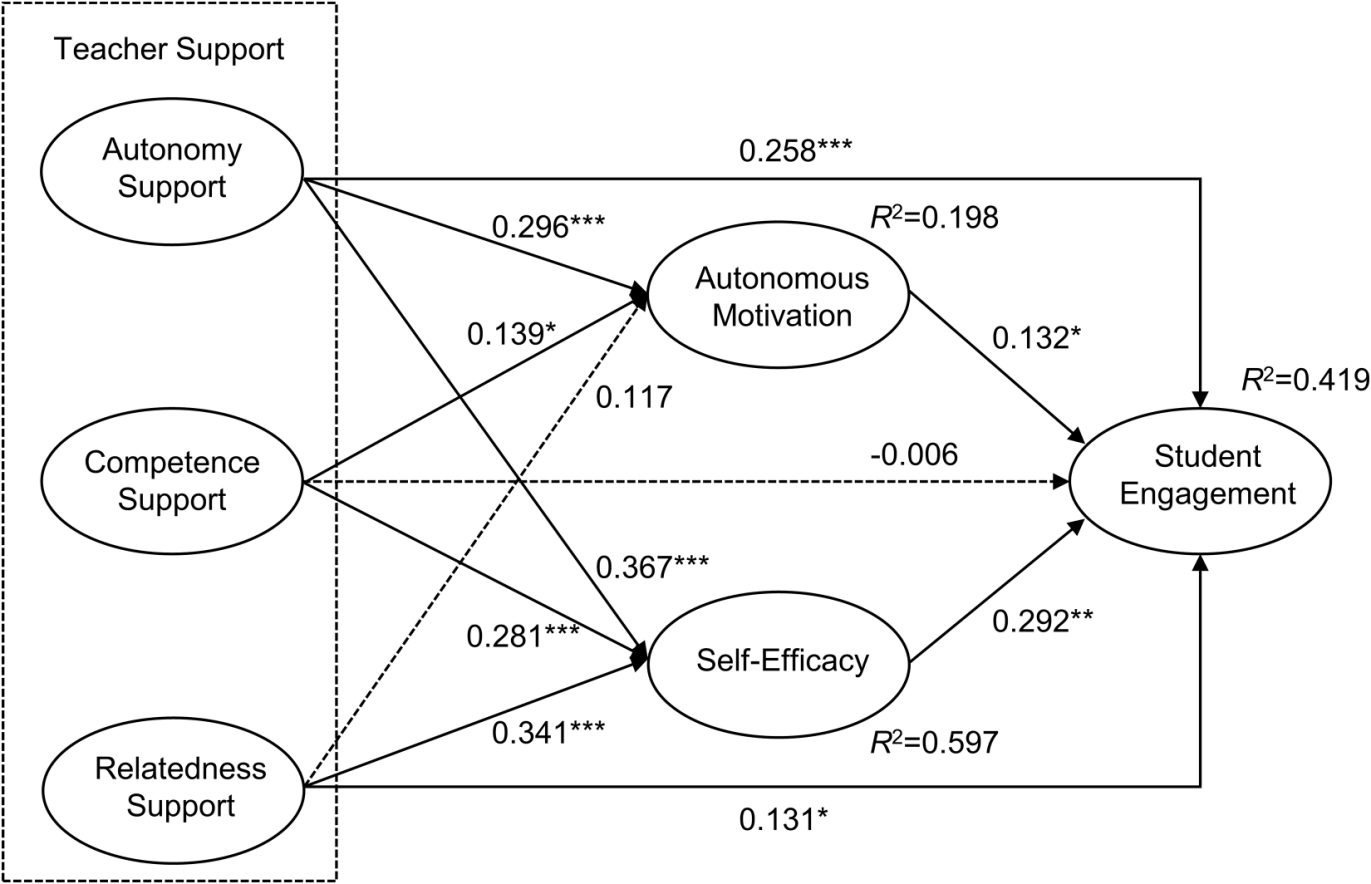
Mediation model with autonomous motivation and self-efficacy as mediators between teacher support and student engagement. Note: **p < 0.01; ***p < 0.001.

The researchers evaluated the hypotheses through structural equation modelling using AMOS 26.0 in this study (Fig 3). The structural model’s fitting indices were all above standard cutoff values, suggesting an overall satisfactory model fit (χ^2^/df = 2.083, SRMR = 0.0592, RMSEA = 0.052, CFI = 0.927, TLI = 0.921, IFI = 0.928).

Table 4 displays the findings from the path analysis. Autonomy support (*β *= 0.258, *p* < 0.001), relatedness support (*β* = 0.131, *p* < 0.05), autonomous motivation (*β* = 0.132, *p* < 0.05), and self-efficacy *(β* = 0.292, *p* < 0.05) were positively correlated with student engagement, supporting H1a and H1c. However, competence support (*β* = −0.006, *p* > 0.05) had no significant effect on student engagement, and H1b was not supported. In addition, autonomy support (*β* = 0.296, *p* < 0.05) and competence support (*β* = 0.139, *p* < 0.05) both positively predicted autonomous motivation. The relationship between relatedness support (*β* = 0.117, *p* > 0.05) and autonomous motivation was insignificant. Moreover, autonomy support (*β* = 0.367, *p* < 0.05), competence support (*β* = 0.281, *p* < 0.05), and relatedness support (*β* = 0.341, *p* < 0.05) were all positively related to self-efficacy.

**Table 4:** Results of path analysis.

Path	Std. (β)	S.E.	Z	*p*
AS→SE	0.258	0.032	3.808	***
CS → SE	−0.006	0.030	−.096	0.924
RS → SE	0.131	0.032	2.038	0.042
AS→AM	0.296	0.049	4.715	***
CS → AM	0.139	0.054	2.076	0.038
RS → AM	0.117	0.052	1.882	0.060
AM → SE	0.132	0.035	2.265	0.024
AS→GSE	0.367	0.045	7.484	***
CS → GSE	0.281	0.046	5.891	***
RS → GSE	0.341	0.048	7.046	***
GSE → SE	0.292	0.045	3.240	0.001

***p < 0.001; AS, Autonomy Support; CS, Competence Support; RS, Relatedness Support; GSE, General Self-Efficacy; AM, Autonomous Motivation; SE, Student Engagement.

To better interpret the results, Cohen [94] suggested that squared multiple correlations (*R*^2^) values of 0.01, 0.09 and 0.25 could be used as thresholds to demonstrate predictive power. As for the explained variance, the proposed model presented good *R*^2^ values for the dependent variables: student engagement (*R*^2 ^= 0.419), self-efficacy (*R*^2 ^= 0.597), and autonomous motivation (*R*^2 ^= 0.198). These results suggested that relatively large impacts of exogenous variables on the endogenous variables.

The bias-corrected percentile bootstrap in AMOS 26.0 software was further used to test the mediation effect (sampled 5,000 times), and 95% confidence intervals were derived accordingly. As shown in Table 5, autonomous motivation had two indirect paths with 95% CI that did not include 0. In particular, autonomous motivation played a mediating role in linking autonomy support (effect = 0.024, Boot CI = [0.0018, 0.0738]) and competence support (effect* *= 0.009, Boot CI = [0.0001, 0.0350]) to student engagement. H2a and H2b were supported. The indirect pathway involving autonomous motivation between relatedness support and student engagement was insignificant (effect = 0.008, Boot CI = [−0.0010, 0.0321]). Thus, H2c was not supported. The 95% CI for all three indirect paths of self-efficacy did not contain 0, indicating that autonomy support (effect = 0.065, Boot CI = [0.0184, 0.1559]), competence support (effect = 0.040, Boot CI = [0.0124, 0.0958]), and relatedness support (effect = 0.050, Boot CI = [0.0141, 0.1095]) exerted indirect effects on student engagement via self-efficacy. Therefore, H3a to H3c were supported.

**Table 5:** Results of mediating effects.

Mediation Path	Effect	Boot SE	*p*	95% Boot CI	
**Lower**	**Upper**
AS→AM → SE	0.024*	0.018	0.032	0.0018	0.0738
CS → AM → SE	0.009*	0.008	0.046	0.0001	0.0350
RS → AM → SE	0.008	0.008	0.072	−0.0010	0.0321
AS→GSE → SE	0.065**	0.034	0.003	0.0184	0.1559
CS → GSE → SE	0.040**	0.020	0.003	0.0124	0.0958
RS → GSE → SE	0.050**	0.024	0.004	0.0141	0.1095

**p < 0.01, *p < 0.05; AS, Autonomy Support; CS, Competence Support; RS, Relatedness Support; GSE, General Self-Efficacy; AM, Autonomous Motivation; SE, Student Engagement.

## 5 Discussion

Under SDT’s theoretical lens, teacher support is regarded as a critical element in fostering student engagement, as it enables the fulfillment of learners’ psychological needs in classroom environments [95]. Nonetheless, the applicability of this association in the more socially interactive PE context has not been fully validated. Rather than considering the multidimensional nature of teacher support, most previous studies have concentrated on a single component, such as autonomy or relatedness support [23]. Hence, grounded in a comprehensive perspective of SDT, this study systematically examined autonomy, competence, and relatedness support to elucidate the underlying mechanisms by which teacher support affects student engagement within PE settings. Additionally, this study developed a parallel mediation model informed by SCT to investigate the potential mediating roles of self-efficacy and autonomous motivation.

### Teacher support and student engagement

Model outcomes indicate that autonomy and relatedness support were closely related to student engagement, with autonomy support identified as the most influential predictor. Consistent with past research in different educational settings [43,96], teachers providing autonomy support in PE can create adaptive environments that grow student engagement. Autonomy-supportive teachers encourage students to think exploratively and build their own experiences during motor skill acquisition rather than forcing them to follow instructions. An autonomy-supportive climate during class is one of the prerequisites for students’ self-determined behavior [97]. When PE is structured in a way that supports autonomy, students display more active engagement, as it enhances their decision-making and the development of fosters deeper processing of information and learning initiative [98]. Furthermore, relatedness support positively predicts college students’ engagement. Relatedness-supported teachers who show enthusiasm and listen to student expressions during PE lessons will contribute to student engagement [49]. In other words, students’ feelings of caring and respect in their interactions with PE teachers can facilitate their emotional experiences in class, thereby increasing their engagement.

Notably, the direct path from competence support to student engagement was insignificant, contrasting with previous studies [20,36]. A possible explanation for this is that there may be a bias in the teaching support behaviour of PE teachers during the course; that is, there is an unconscious tendency to provide more competence support to more capable students [99]. Due to the demonstrative nature of PE classes, compared to other subjects, differences in students’ abilities are more likely to manifest. In this context, the less experienced students receive insufficient support for teacher competence, which may lead to a state of learned helplessness in their PE classes. Another possible reason is that college PE teachers in China may tend towards repetitiveness and simplicity in the structural arrangement of course content, and this way of instruction struggles to adapt to the varied needs of college students [100]. This rigid teaching pattern may make it difficult for students to obtain timely feedback on their learning. Even if students report a certain level of perceived competence support, they may not benefit from it.

Alongside teacher-related considerations, both student and contextual conditions merit attention in understanding this finding. Students with lower prior competence perceptions may hold weaker ability beliefs, which makes it harder for them to benefit directly from competence support, particularly when such support is insufficiently aligned with their personal learning characteristics. At the contextual level, constraints such as large class sizes and limited sports facilities may further reduce opportunities for personalized guidance, thereby hindering the direct translation of perceived competence support into active engagement in PE classes. Therefore, college PE teachers should focus on individual differences, implement tiered or personalized instruction, provide targeted feedback, and help students develop a sense of achievement through appropriate learning goals, thus promoting long-term student engagement. As evidenced by a study focusing on Asian student engagement, even those students who were disheartened and with low perceived physical ability recognized the value of PE teachers’ encouragement and skill-focused feedback in achieving success [101].

### The mediating role of autonomous motivation

The mediation analysis results demonstrate that autonomous motivation acted as a mediator linking autonomy support to student engagement in PE, which corresponds to previous findings. Yoo [47] also found that autonomous motivation mediated the association between autonomy support and student behavioral engagement. SDT emphasizes that autonomy support fosters more self-determined and internally regulated forms of motivation [17]. Autonomous motivation is more likely to emerge in autonomy-supportive environments, where teachers encourage students, value their perspectives, and demonstrate patience. Thus, teacher autonomy support enables students to have stronger autonomous motivation, making them more inclined to tackle learning-related challenges and develop sustained engagement [30,60]. In addition, the findings also showed that competence support influenced student engagement indirectly through autonomous motivation. Competence support from teachers can stimulate autonomous motivation and lead students to place greater value on PE lessons [102]. Students who recognize that teachers offer effective instruction, provide clear expectations, and show concern for their progress tend to exhibit higher autonomous motivation [103]. Elevated autonomous motivation helps enhance student engagement throughout PE sessions.

Contrary to our expectations, the pathway from relatedness support to student engagement via autonomous motivation was not significant. This finding does not align with previous research [56]. At the statistical level, this outcome is due to the non-significant association between relatedness support and autonomous motivation in this study. A possible explanation is that Chinese college students typically change PE teachers after each semester due to adjustments in the motor learning curriculum, which may result in insufficient external conditions that hinder the development of constructive bonds between teachers and students. According to SDT, relatedness support can effectively promote the internalization of motivation only when it satisfies individuals’ need for relatedness and facilitates the identification and integration of external assigned goal values [104]. In contexts where instructors are frequently replaced, students may struggle to develop stable perceptions of relatedness, thereby diminishing the positive impact of relatedness support on autonomous motivation. Notably, Slemp et al. [105] indicated that cultural context moderates the association between relatedness support and intrinsic motivation, with stronger effects observed in more individualistic cultures. Although collectivist cultures emphasize interpersonal harmony and social connectedness, institutional constraints such as a lack of continuity in teacher–student relationships may lead students to interpret relatedness support as a fulfillment of professional duties or social expectations rather than as an expression of genuine care from educators. Under these circumstances, student engagement in PE tends to be determined by external motivations such as credit requirements, grade evaluation, or avoidance of punishment. Future educational practices could create more adequate conditions for linking teachers’ relatedness support with autonomous motivation by lengthening the interaction cycle, increasing informal interactions (e.g., after-school exchanges), and designing collaborative learning scenarios.

### The mediating role of self-efficacy

Results from this research confirmed that self-efficacy is a mediating variable linking autonomy support to student engagement. In line with previous empirical work, when teachers help students perceive self-determination in their behavior, the autonomy learning climate reinforces students’ affirmation of their abilities, which improves their self-efficacy [25]. A sense of self-efficacy makes students feel more confident in independently completing learning tasks, which promotes engagement. Teachers can provide autonomy support by offering meaningful choices and advocating the value of learning, thereby increasing students’ self-efficacy and indirectly improving student engagement [106]. In addition, the impact of competence support on student engagement is mediated by self-efficacy. This implies that students who perceive clear goals and guidance from PE teachers can possess strong self-efficacy, enabling them to overcome challenges in learning motor skills. Similarly, Zhen et al. [26] noted that teachers’ competence support increases students’ beliefs in reaching learning goals, thus enhancing students’ engagement. Moreover, the results reveal the mediating role of self-efficacy in the relation of relatedness support with student engagement among college students. Teacher relatedness support is reflected in encouraging, listening, and fair treatment of students. This type of support by teachers helps to develop an inclusive and trusting relationship with the students and enhances their self-efficacy. A heightened sense of self-efficacy among students generally leads to greater effort in completing assigned learning tasks, and they are inclined to be engaged during the lesson [67,107]. To sum up, helping students develop self-efficacy is an essential target of teacher support, and it is also a key strategy to inspire them to be more engaged in PE.

### Theoretical and practical implications

The present study explores teacher support across three dimensions to clarify how each form of support influences the regulation of student motivation and behavior. The results provide empirical support for the core hypotheses of SDT. Specifically, PE teachers’ autonomy and relatedness support predicted student engagement, while autonomy and competence support were positively related to autonomous motivation. Our investigation validates the significant impact of psychological need support on student motivation and behavior, reinforcing the SDT perspective that motivation arises dynamically from the interplay of an individual’s psychological needs and environmental conditions rather than a singular internal inclination. Furthermore, by validating the findings among Chinese college students in a collectivist culture, this study provides additional evidence supporting the cross-cultural generalizability of SDT and SCT. Self-efficacy was shown to function as a key mediator in this study, lending support to the Triadic Reciprocal Determinism in SCT. This reveals the dynamic interaction mechanism among environmental conditions (teacher support), personal attributes (self-efficacy), and behaviors (student engagement), which provides important theoretical and empirical evidence to improve and optimize the interaction of teaching in higher education PE. Furthermore, autonomous motivation was identified as a mediator, revealing how PE teacher support promotes student engagement through internal motivational processes. Taken together, these results not only deepen our understanding of how teacher support contributes to student engagement but also offer an integrative framework for tackling the problem of inadequate engagement in college PE classes.

This study underscores the value of teacher support in enhancing student engagement in physical education. PE teachers in higher education should promote autonomy support by providing students with decision-making opportunities (e.g., student-led warm-up sessions), incorporating student interests into instructional content, and emphasizing the value of motor skill learning. In addition, they can foster competence support by conveying clear expectations for skill learning, dynamically adjusting their teaching strategies and goals to match students’ varying proficiency levels (e.g., differentiated instruction), and providing timely feedback alongside recognition of their progress. Relatedness support can also be cultivated by strengthening the teacher–student emotional connection through respect and care, consistently providing unconditional attention, and encouraging students to engage in cooperative tasks (e.g., team-based challenges). Second, teachers must dedicate efforts to developing students’ self-efficacy and autonomous motivation in PE, as these are critical pathways to enhance student engagement. Specifically, cultivating students’ self-efficacy involves teachers conveying reasonable goals, trust, and positive feedback to students, assisting them in gaining more successful experiences. PE teachers should also adopt in affirming interpersonal communication to help students form positive efficacy expectations, strengthen their capacity for emotional regulation, and develop cognitive flexibility, thereby fostering greater engagement in motor skill learning. The cultivation of students’ autonomous motivation emphasizes the internalization of motivation. Therefore, teachers should combine supportive teaching to create a motivational learning climate, encourage students to think and solve problems independently, and ultimately let them realize the value of PE learning to stimulate autonomous motivation. In this process, teachers should avoid over-relying on external incentives such as course credits or punishment. A more effective approach is to guide students to understand the significance of physical activity based on their own interests and goals, thereby facilitating the gradual internalization of external motivation into autonomous motivation. In addition, whether teachers’ supportive teaching can be implemented is affected to a certain extent by their working environment. Accordingly, educational administrators should establish a flexible course implementation system to guide teachers in optimizing instructional design. Building on this, teachers can be encouraged to incorporate instructional strategies such as group-based teaching and collaborative learning in class, thereby creating conditions for more tailored guidance, timely feedback, and in-depth interaction that stimulate students’ learning initiative. Furthermore, instructional pacing and objectives should be progressively adjusted according to students’ physical fitness levels and feedback, enabling a more precise identification and addressing of diverse learner needs. To further enhance the effectiveness of these strategies, regular teaching seminars can be organized to share best practices. Finally, colleges can provide training programs for PE teachers on motivational instruction and integrate these contents into student evaluations, thereby promoting teachers’ self-examination, self-reflection, and self-improvement. In sum, these measures help teachers raise awareness of supporting students and develop quality supportive PE teaching practices.

## 6 Limitations and future directions

Given time and resource constraints, this study conducted questionnaire surveys only in four colleges in Jiangxi Province, China. As a result, contextual variations among Chinese higher education institutions may limit the broader applicability of the findings, as students in other regions may exhibit different characteristics, experiences, or perspectives. Future research could employ a multi-site or nationally representative sampling strategy to improve the external validity and generalizability of the findings.

Our research employed a cross-sectional methodology, which allows for capturing the complex student engagement process at a particular time. However, the nature of the cross-sectional approach may limit the inference of causality. Future longitudinal and experimental studies may help capture the dynamics and developmental trajectories of student engagement, revealing causal relationships.

Despite presenting an integrated model predicting student engagement in physical education, this study did not assess measurement invariance across subgroups with diverse characteristics (e.g., gender, academic year). Testing measurement and structural invariance in future research with larger samples would be valuable for verifying whether the proposed model operates equivalently across varied student subpopulations and cultural contexts.

Finally, this study measured student engagement using self-reported data, and the findings may be subject to participants’ interpretations of the questionnaire items and potential response biases (e.g., social desirability and recall bias). To minimize this limitation, the purpose and significance of the questionnaire were explained in detail before distribution, and the anonymity of the answers was emphasized to respondents. Future research could consider adopting an external observer’s scoring approach or using wearable exercise tracking devices to monitor students’ physical responses during PE lessons, ensuring higher measurement precision.

## 7 Conclusion

Based on SDT and SCT, this study examined the mechanisms underlying how teacher support (autonomy, competence, and relatedness support) influences student engagement within the PE context. The study revealed that teacher autonomy and relatedness support directly influenced student engagement. Autonomous motivation mediated the effects of both autonomy and competence support on student engagement. Self-efficacy was a crucial mediator across all three pathways linking teacher support (autonomy, competence, and relatedness) with student engagement. Overall, this study reveals potential pathways linking teacher support and student engagement among college students in PE settings, laying the groundwork for subsequent instructional interventions and practical strategies aimed at promoting sustained student engagement.

## Supporting information

S1 File Data. (XLSX)

S2 File Inclusivity in global research. (DOCX)

## References

[1] Prananto K, Cahyadi S, Lubis FY, Hinduan ZR. Perceived teacher support and student engagement among higher education students - a systematic literature review. BMC Psychol. 2025;13(1):112. doi: 10.1186/s40359-025-02412-w 39934874 PMC11817619

[2] Luo Q, Chen L, Yu D, Zhang K. The Mediating Role of Learning Engagement Between Self-Efficacy and Academic Achievement Among Chinese College Students. Psychol Res Behav Manag. 2023;16:1533–43. doi: 10.2147/PRBM.S401145 37143904 PMC10153452

[3] Tinto V. Exploring the Character of Student Persistence in Higher Education: The Impact of Perception, Motivation, and Engagement. Handbook of Research on Student Engagement. Springer International Publishing. 2022. p. 357–79. doi: 10.1007/978-3-031-07853-8_17

[4] Salmela-Aro K, Tang X, Upadyaya K. Study Demands-Resources Model of Student Engagement and Burnout. Handbook of Research on Student Engagement. Springer International Publishing. 2022. p. 77–93. doi: 10.1007/978-3-031-07853-8_4

[5] Reschly AL. Dropout Prevention and Student Engagement. Student Engagement. Springer International Publishing. 2020. p. 31–54. doi: 10.1007/978-3-030-37285-9_2

[6] Passeggia R, Testa I, Esposito G, Picione RDL, Ragozini G, Freda MF. Examining the Relation Between First-year University Students’ Intention to Drop-out and Academic Engagement: The Role of Motivation, Subjective Well-being and Retrospective Judgements of School Experience. Innov High Educ. 2023;48(5):837–59. doi: 10.1007/s10755-023-09674-5

[7] Standage M, Ryan RM, Curran T. Self-Determination Theory Applied to Physical Education: The Role of Self-Regulatory Processes in Facilitating High-Quality Student Motivation, Engagement, and Well-Being. Motivation in Physical Education. Springer Nature Switzerland. 2025. p. 29–51. doi: 10.1007/978-3-031-86908-2_2

[8] Maloshonok N. Do student engagement patterns differ across national higher education systems? The comparison of US, Chinese, and Russian high-level research-intensive universities. Innovations in Education and Teaching International. 2023;61(3):475–86. doi: 10.1080/14703297.2023.2183883

[9] Bianchi N. The Indirect Effects of Educational Expansions: Evidence from a Large Enrollment Increase in University Majors. Journal of Labor Economics. 2020;38(3):767–804. doi: 10.1086/706050

[10] Noetel M, Parker P, Dicke T, Beauchamp MR, Ntoumanis N, Hulteen RM, et al. Prediction Versus Explanation in Educational Psychology: a Cross-Theoretical Approach to Using Teacher Behaviour to Predict Student Engagement in Physical Education. Educ Psychol Rev. 2023;35(3). doi: 10.1007/s10648-023-09786-6

[11] Zhu Q, Jia L. Research on the path of constructing immersive public physical education classes in universities based on individual sensory channels. Liaoning Sport Science and Technology. 2025;47:114–9. doi: 10.13940/j.cnki.lntykj.2025.04.001

[12] Liu L, Li G. The Dilemma and Optimization Path of the Individualization of Public Physical Education Teaching in Universities—Based on the Dreyfus Skill Acquisition Model. Journal of Nanjing Sports Institute. 2024;23:61–4, 71. doi: 10.15877/j.cnki.nsin.2024.08.007

[13] Li Y, Lerner RM. Trajectories of school engagement during adolescence: implications for grades, depression, delinquency, and substance use. Dev Psychol. 2011;47(1):233–47. doi: 10.1037/a0021307 21244162

[14] Lawson H, Lawson M. Student Engagement and Disengagement as a Collective Action Problem. Education Sciences. 2020;10(8):212. doi: 10.3390/educsci10080212

[15] Cleland CL, Tully MA, Kee F, Cupples ME. The effectiveness of physical activity interventions in socio-economically disadvantaged communities: a systematic review. Prev Med. 2012;54(6):371–80. doi: 10.1016/j.ypmed.2012.04.004 22521997

[16] Wong ZY, Liem GAD. Student Engagement: Current State of the Construct, Conceptual Refinement, and Future Research Directions. Educ Psychol Rev. 2021;34(1):107–38. doi: 10.1007/s10648-021-09628-3

[17] Haerens L, Aelterman N, Vansteenkiste M, Soenens B, Van Petegem S. Do perceived autonomy-supportive and controlling teaching relate to physical education students’ motivational experiences through unique pathways? Distinguishing between the bright and dark side of motivation. Psychology of Sport and Exercise. 2015;16:26–36. doi: 10.1016/j.psychsport.2014.08.013

[18] Kaplan H. Teachers’ autonomy support, autonomy suppression and conditional negative regard as predictors of optimal learning experience among high-achieving Bedouin students. Soc Psychol Educ. 2017;21(1):223–55. doi: 10.1007/s11218-017-9405-y

[19] Patall EA, Vite A, Lee DJ, Zambrano J. Teacher support for students’ psychological needs and student engagement: Differences across school levels based on a national teacher survey. Teaching and Teacher Education. 2024;137:104400. doi: 10.1016/j.tate.2023.104400

[20] Otundo JO, Garn AC. Student Interest and Engagement in Middle School Physical Education: Examining the Role of Needs Supportive Teaching. IJEP. 2019;8(2):137. doi: 10.17583/ijep.2019.3356

[21] Coterón J, Franco E, Ocete C, Pérez-Tejero J. Teachers’ Psychological Needs Satisfaction and Thwarting: Can They Explain Students’ Behavioural Engagement in Physical Education? A Multi-Level Analysis. Int J Environ Res Public Health. 2020;17(22):8573. doi: 10.3390/ijerph17228573 33227917 PMC7699264

[22] Sun J, Wang J, Wang S. Improving the comprehensive literacy of physical education teachers in universities under the guidance of the spirit of educators: Theoretical interpretation and practical approach. Journal of Beijing Sport University. 2024;47:89–99. doi: 10.19582/j.cnki.11-3785/g8.2024.10.011

[23] Guo Q, Samsudin S, Yang X, Gao J, Ramlan MA, Abdullah B, et al. Relationship between Perceived Teacher Support and Student Engagement in Physical Education: A Systematic Review. Sustainability. 2023;15(7):6039. doi: 10.3390/su15076039

[24] Lo Schiavo M, Prinari B, Saito I, Shoji K, Benight CC. A dynamical systems approach to triadic reciprocal determinism of social cognitive theory. Mathematics and Computers in Simulation. 2019;159:18–38. doi: 10.1016/j.matcom.2018.10.006

[25] Li W, Gao W, Sha J. Perceived Teacher Autonomy Support and School Engagement of Tibetan Students in Elementary and Middle Schools: Mediating Effect of Self-Efficacy and Academic Emotions. Front Psychol. 2020;11:50. doi: 10.3389/fpsyg.2020.00050 32082219 PMC7005053

[26] Zhen R, Liu R-D, Ding Y, Wang J, Liu Y, Xu L. The mediating roles of academic self-efficacy and academic emotions in the relation between basic psychological needs satisfaction and learning engagement among Chinese adolescent students. Learning and Individual Differences. 2017;54:210–6. doi: 10.1016/j.lindif.2017.01.017

[27] Azila-Gbettor EM, Mensah C, Abiemo MK, Bokor M. Predicting student engagement from self-efficacy and autonomous motivation: A cross-sectional study. Cogent Education. 2021;8(1). doi: 10.1080/2331186x.2021.1942638

[28] Chuang KL, Kee YH, Chen HH. Implementation of the gradual release of responsibility informed curriculum and pedagogy for teaching programming: Action research based on a course for sport science students. Journal of Hospitality, Leisure, Sport & Tourism Education. 2022;30:100367. doi: 10.1016/j.jhlste.2021.100367

[29] Ryan RM, Deci EL. Self-Determination Theory: Basic Psychological Needs in Motivation, Development, and Wellness. Guilford Publications. 2017.

[30] Noels KA, Vargas Lascano DI, Saumure K. The development of self-determination across the language course. Stud Second Lang Acquis. 2019;41(04):821–51. doi: 10.1017/s0272263118000189

[31] Haw JY, King RB. Perceived need-supportive leadership, perceived need-supportive teaching, and student engagement: A self-determination perspective. Soc Psychol Educ. 2023;26(5):1289–319. doi: 10.1007/s11218-023-09790-2

[32] Cheng W. How intrinsic and extrinsic motivations function among college student samples in both Taiwan and the U.S. Educational Psychology. 2018;39(4):430–47. doi: 10.1080/01443410.2018.1510116

[33] Urhahne D, Wijnia L. Theories of Motivation in Education: an Integrative Framework. Educ Psychol Rev. 2023;35(2). doi: 10.1007/s10648-023-09767-9

[34] Bureau JS, Howard JL, Chong JXY, Guay F. Pathways to Student Motivation: A Meta-Analysis of Antecedents of Autonomous and Controlled Motivations. Rev Educ Res. 2022;92(1):46–72. doi: 10.3102/00346543211042426 35330866 PMC8935530

[35] Ye J, Zhang LJ, Dixon H. Reconceptualising student feedback agency from a social cognitive perspective. Assessment in Education: Principles, Policy & Practice. 2025;1–16. doi: 10.1080/0969594x.2025.2533122

[36] Leo FM, Pulido JJ, Sánchez-Oliva D, López-Gajardo MA, Mouratidis A. See the forest by looking at the trees: Physical education teachers’ interpersonal style profiles and students’ engagement. European Physical Education Review. 2022;28(3):720–38. doi: 10.1177/1356336x221075501

[37] Deci EL, Ryan RM. The “What” and “Why” of Goal Pursuits: Human Needs and the Self-Determination of Behavior. Psychological Inquiry. 2000;11(4):227–68. doi: 10.1207/s15327965pli1104_01

[38] Reeve J. A Self-determination Theory Perspective on Student Engagement. In: Christenson SL, Reschly AL, Wylie C, Editors. Handbook of Research on Student Engagement. Boston, MA: Springer US; 2012. pp. 149–172. doi: 10.1007/978-1-4614-2018-7_7

[39] Liu H, Li X. Unravelling students’ perceived EFL teacher support. System. 2023;115:103048. doi: 10.1016/j.system.2023.103048

[40] Ahmadi A, Noetel M, Parker P, Ryan RM, Ntoumanis N, Reeve J, et al. A classification system for teachers’ motivational behaviors recommended in self-determination theory interventions. Journal of Educational Psychology. 2023;115(8):1158–76. doi: 10.1037/edu0000783

[41] Olivier E, Galand B, Morin AJ, Hospel V. Need-supportive teaching and student engagement in the classroom: Comparing the additive, synergistic, and global contributions. Learn Instr. 2021;71: 101389. doi: 10.1016/j.learninstruc.2020.101389

[42] Chiu TKF. Applying the self-determination theory (SDT) to explain student engagement in online learning during the COVID-19 pandemic. Journal of Research on Technology in Education. 2021;54(sup1). doi: 10.1080/15391523.2021.1891998

[43] Patall EA, Steingut RR, Vasquez AC, Trimble SS, Pituch KA, Freeman JL. Daily autonomy supporting or thwarting and students’ motivation and engagement in the high school science classroom. Journal of Educational Psychology. 2018;110(2):269–88. doi: 10.1037/edu0000214

[44] Fried L, Konza D. Using Self-Determination Theory to Investigate Student Engagement in the Classroom. The International Journal of Pedagogy and Curriculum. 2013;19(2):27–40. doi: 10.18848/2327-7963/cgp/v19i02/48898

[45] Cents-Boonstra M, Lichtwarck-Aschoff A, Denessen E, Aelterman N, Haerens L. Fostering student engagement with motivating teaching: an observation study of teacher and student behaviours. Research Papers in Education. 2020;36(6):754–79. doi: 10.1080/02671522.2020.1767184

[46] De Meyer J, Soenens B, Vansteenkiste M, Aelterman N, Van Petegem S, Haerens L. Do students with different motives for physical education respond differently to autonomy-supportive and controlling teaching?. Psychology of Sport and Exercise. 2016;22:72–82. doi: 10.1016/j.psychsport.2015.06.001

[47] Yoo J. Perceived autonomy support and behavioral engagement in physical education: a conditional process model of positive emotion and autonomous motivation. Percept Mot Skills. 2015;120(3):731–46. doi: 10.2466/06.PMS.120v20x8 26057419

[48] Shen B, McCaughtry N, Martin JJ, Fahlman M, Garn AC. Urban High-School Girls’ Sense of Relatedness and Their Engagement in Physical Education. Journal of Teaching in Physical Education. 2012;31(3):231–45. doi: 10.1123/jtpe.31.3.231

[49] González-Peño A, Franco E, Coterón J. Do Observed Teaching Behaviors Relate to Students’ Engagement in Physical Education?. Int J Environ Res Public Health. 2021;18(5):2234. doi: 10.3390/ijerph18052234 33668255 PMC7967671

[50] Zhang BG, Qian XF. Perceived teacher’s support and engagement among students with obesity in physical education: The mediating role of basic psychological needs and autonomous motivation. J Sports Sci. 2022;40(17):1901–11. doi: 10.1080/02640414.2022.2118935 36062925

[51] Wang C, Cho HJ, Wiles B, Moss JD, Bonem EM, Li Q, et al. Competence and autonomous motivation as motivational predictors of college students’ mathematics achievement: from the perspective of self-determination theory. IJ STEM Ed. 2022;9(1). doi: 10.1186/s40594-022-00359-7

[52] Guay F. Applying Self-Determination Theory to Education: Regulations Types, Psychological Needs, and Autonomy Supporting Behaviors. Canadian Journal of School Psychology. 2021;37(1):75–92. doi: 10.1177/08295735211055355

[53] Paumier D, Chanal J. The antecedents and consequences of autonomous and controlled motivation: Domain specificity and motivational sequence at the situational level. Front Psychol. 2022;13:987582. doi: 10.3389/fpsyg.2022.987582 36248442 PMC9559569

[54] Ryan RM, Deci EL. Intrinsic and extrinsic motivation from a self-determination theory perspective: Definitions, theory, practices, and future directions. Contemporary Educational Psychology. 2020;61:101860. doi: 10.1016/j.cedpsych.2020.101860

[55] Abula K, Beckmann J, He Z, Cheong C, Lu F, Gröpel P. Autonomy support in physical education promotes autonomous motivation towards leisure-time physical activity: evidence from a sample of Chinese college students. Health Promot Int. 2020;35(1):e1–10. doi: 10.1093/heapro/day102 30590612

[56] Gairns F, Whipp PR, Jackson B. Relational perceptions in high school physical education: teacher- and peer-related predictors of female students’ motivation, behavioral engagement, and social anxiety. Front Psychol. 2015;6:850. doi: 10.3389/fpsyg.2015.00850 26157404 PMC4475790

[57] Ganotice FA Jr, Chan CS, Chan EWY, Chan SKW, Chan L, Chan SCS, et al. Autonomous motivation predicts students’ engagement and disaffection in interprofessional education: Scale adaptation and application. Nurse Educ Today. 2022;119:105549. doi: 10.1016/j.nedt.2022.105549 36182789

[58] Leo FM, Mouratidis A, Pulido JJ, López-Gajardo MA, Sánchez-Oliva D. Perceived teachers’ behavior and students’ engagement in physical education: the mediating role of basic psychological needs and self-determined motivation. Physical Education and Sport Pedagogy. 2020;27(1):59–76. doi: 10.1080/17408989.2020.1850667

[59] Yang Y, Du C. The predictive effect of perceived teacher support on college EFL learners’ online learning engagement: autonomous and controlled motivation as mediators. Journal of Multilingual and Multicultural Development. 2023;:1–15. doi: 10.1080/01434632.2023.2259879

[60] Polet J, Lintunen T, Schneider J, Hagger MS. Predicting change in middle school students’ leisure‐time physical activity participation: A prospective test of the trans‐contextual model. J Applied Social Pyschol. 2020;50(9):512–23. doi: 10.1111/jasp.12691

[61] Mynard J, Shelton-Strong SJ. Autonomy Support Beyond the Language Learning Classroom. Channel View Publications. 2022. doi: 10.2307/jj.22679762

[62] Burgueño R, García-González L, Abós Á, Sevil-Serrano J. Students’ motivational experiences across profiles of perceived need-supportive and need-thwarting teaching behaviors in physical education. Physical Education and Sport Pedagogy. 2022;29(1):82–96. doi: 10.1080/17408989.2022.2028757

[63] Diloy-Peña S, García-González L, Burgueño R, Tilga H, Koka A, Abós Á. A Cross-Cultural Examination of the Role of (De-)Motivating Teaching Styles in Predicting Students’ Basic Psychological Needs in Physical Education: A Circumplex Approach. Journal of Teaching in Physical Education. 2025;44(2):272–84. doi: 10.1123/jtpe.2023-0036

[64] Vasconcellos D, Parker PD, Hilland T, Cinelli R, Owen KB, Kapsal N, et al. Self-determination theory applied to physical education: A systematic review and meta-analysis. Journal of Educational Psychology. 2020;112(7):1444–69. doi: 10.1037/edu0000420

[65] Ryan RM, Duineveld JJ, Di Domenico SI, Ryan WS, Steward BA, Bradshaw EL. We know this much is (meta-analytically) true: A meta-review of meta-analytic findings evaluating self-determination theory. Psychological Bulletin. 2022;148(11–12):813–42. doi: 10.1037/bul0000385

[66] Patzak A, Zhang X. Blending Teacher Autonomy Support and Provision of Structure in the Classroom for Optimal Motivation: A Systematic Review and Meta-Analysis. Educ Psychol Rev. 2025;37(1). doi: 10.1007/s10648-025-09994-2

[67] Sökmen Y. The role of self-efficacy in the relationship between the learning environment and student engagement. Educational Studies. 2019;47(1):19–37. doi: 10.1080/03055698.2019.1665986

[68] Bartimote-Aufflick K, Bridgeman A, Walker R, Sharma M, Smith L. The study, evaluation, and improvement of university student self-efficacy. Studies in Higher Education. 2015;41(11):1918–42. doi: 10.1080/03075079.2014.999319

[69] Bandura A. On the Functional Properties of Perceived Self-Efficacy Revisited. Journal of Management. 2011;38(1):9–44. doi: 10.1177/0149206311410606

[70] Zhou M, Liu X, Guo J. The mediating effect of self-efficacy between teacher emotional support and interaction engagement in EFL learning. Journal of Multilingual and Multicultural Development. 2023;:1–15. doi: 10.1080/01434632.2023.2267033

[71] Hu Z, Shan N, Jiao R. The relationships between perceived teacher autonomy support, academic self-efficacy and learning engagement among primary school students: A network analysis. Eur J Psychol Educ. 2023;39(2):503–16. doi: 10.1007/s10212-023-00703-7

[72] Duchatelet D, Donche V. Fostering self-efficacy and self-regulation in higher education: a matter of autonomy support or academic motivation?. Higher Education Research & Development. 2019;38(4):733–47. doi: 10.1080/07294360.2019.1581143

[73] Olivier E, Archambault I, De Clercq M, Galand B. Student Self-Efficacy, Classroom Engagement, and Academic Achievement: Comparing Three Theoretical Frameworks. J Youth Adolesc. 2019;48(2):326–40. doi: 10.1007/s10964-018-0952-0 30421327

[74] Lin T-J. Multi-dimensional explorations into the relationships between high school students’ science learning self-efficacy and engagement. International Journal of Science Education. 2021;43(8):1193–207. doi: 10.1080/09500693.2021.1904523

[75] Jia X, Cai L, Lin L, Lin C. The relationship between perceived teachers’ support and academic engagement among high school students: The chain mediating effect of academic self-efficacy and achievement goal orientation. Psychological Development and Education. 2020;36:700–7. doi: 10.16187/j.cnki.issn1001-4918.2020.06.08

[76] Liu Y, Qi H. The necessity and implementation pathways for integrating physical education across primary, secondary, and higher education. China Higher Education. 2022;:49–51.

[77] Brislin RW. Back-Translation for Cross-Cultural Research. Journal of Cross-Cultural Psychology. 1970;1(3):185–216. doi: 10.1177/135910457000100301

[78] Standage M, Duda JL, Ntoumanis N. A test of self-determination theory in school physical education. Br J Educ Psychol. 2005;75(Pt 3):411–33. doi: 10.1348/000709904X22359 16238874

[79] Wilhelmsen T, Sørensen M, Seippel ØN. Motivational Pathways to Social and Pedagogical Inclusion in Physical Education. Adapt Phys Activ Q. 2018;:1–23. doi: 10.1123/apaq.2018-0019 30525925

[80] Skinner E, Furrer C, Marchand G, Kindermann T. Engagement and disaffection in the classroom: Part of a larger motivational dynamic?. Journal of Educational Psychology. 2008;100(4):765–81. doi: 10.1037/a0012840

[81] Wolters CA. Advancing Achievement Goal Theory: Using Goal Structures and Goal Orientations to Predict Students’ Motivation, Cognition, and Achievement. Journal of Educational Psychology. 2004;96(2):236–50. doi: 10.1037/0022-0663.96.2.236

[82] Wilson PM, Rodgers WM, Loitz CC, Scime G. “It’s Who I Am … Really!’ The Importance of Integrated Regulation in Exercise Contexts1. J Appl Biobehavioral Res. 2006;11(2):79–104. doi: 10.1111/j.1751-9861.2006.tb00021.x

[83] Luo Y, Mullin EM, Mellano KT, Sha Y, Wang C. Examining the psychometric properties of the Chinese Behavioral Regulation in Exercise Questionnaire-3: A bi-factor approach. PLoS One. 2022;17(3):e0265004. doi: 10.1371/journal.pone.0265004 35255098 PMC8901058

[84] Schwarzer R, Jerusalem M. Generalized self-efficacy scale. J Weinman Wright M Johnston Meas Health Psychol User’s Portf Causal Control Beliefs. 1995;35:37.

[85] Yang N. The effects of physical activity on college students’ subjective well-being: the mediating role of self-efficacy and the moderating role of exercise achievement motivation. Physical Education Review. 2023.

[86] Wu Y, Wen Z. Item parceling strategies in structural equation modeling. Advances in Psychological Science. 2011;19:1859–67. doi: 10.3724/SP.J.1042.2011.01859

[87] Preacher KJ, Hayes AF. Contemporary Approaches to Assessing Mediation in Communication Research. The SAGE Sourcebook of Advanced Data Analysis Methods for Communication Research. Sage Publications, Inc. 2008. p. 13–54. doi: 10.4135/9781452272054.n2

[88] Podsakoff PM, MacKenzie SB, Lee J-Y, Podsakoff NP. Common method biases in behavioral research: a critical review of the literature and recommended remedies. J Appl Psychol. 2003;88(5):879–903. doi: 10.1037/0021-9010.88.5.879 14516251

[89] Wen Z, Huang B, Tang D. Preliminary work for modeling questionnaire data. Journal of Psychological Science. 2018;41:204–10. doi: 10.16719/j.cnki.1671-6981.20180130

[90] Kock N, Lynn G. Lateral Collinearity and Misleading Results in Variance-Based SEM: An Illustration and Recommendations. JAIS. 2012;13(7):546–80. doi: 10.17705/1jais.00302

[91] Hair JF, Babin BJ, Black WC, Anderson RE. Multivariate Data Analysis. Cengage. 2019.

[92] Fornell C, Larcker DF. Evaluating structural equation models with unobservable variables and measurement error. J Mark Res. 1981;18:39–50.

[93] Henseler J, Ringle CM, Sarstedt M. Testing measurement invariance of composites using partial least squares. International Marketing Review. 2016;33(3):405–31. doi: 10.1108/imr-09-2014-0304

[94] Cohen J. Statistical Power Analysis for the Behavioral Sciences. Routledge. 2013. doi: 10.4324/9780203771587

[95] Reeve J, Shin SH. How teachers can support students’ agentic engagement. Theory Into Practice. 2020;59(2):150–61. doi: 10.1080/00405841.2019.1702451

[96] Miao J, Ma L. Teacher Autonomy Support Influence on Online Learning Engagement: The Mediating Roles of Self-Efficacy and Self-Regulated Learning. Sage Open. 2023;13(4). doi: 10.1177/21582440231217737

[97] Reeve J, Cheon SH, Yu TH. An autonomy-supportive intervention to develop students’ resilience by boosting agentic engagement. International Journal of Behavioral Development. 2020;44(4):325–38. doi: 10.1177/0165025420911103

[98] Meng HY, Keng JWC. The effectiveness of an Autonomy-Supportive Teaching Structure in Physical Education. [Eficacia de la estructura de enseñanza con soporte de autonomía en educación física]. rev int cienc deporte. 2016;12(43):5–28. doi: 10.5232/ricyde2016.04301

[99] Laxdal A, Johannsson E, Giske R. The Role of Perceived Competence in Determining Teacher Support in Upper Secondary School Physical Education. TPE. 2020;77(2):384–403. doi: 10.18666/tpe-2020-v77-i2-9606

[100] He C, Tang W, Tian L. Research on the European and American experience and enlightenment of PE teachers’ teaching style evolution mode in the colleges and universities of China. Sports & Science. 2022;43:61–7. doi: 10.13598/j.issn1004-4590.2022.01.008

[101] Li J, Philpot RA, Bruce T. Asian minds and bodies: a fresh lens on Asian student engagement in physical education. Sport, Education and Society. 2025;:1–14. doi: 10.1080/13573322.2024.2445116

[102] García-González L, Haerens L, Abós Á, Sevil-Serrano J, Burgueño R. Is high teacher directiveness always negative? Associations with students’ motivational outcomes in physical education. Teaching and Teacher Education. 2023;132:104216. doi: 10.1016/j.tate.2023.104216

[103] Mouratidis A, Michou A, Telli S, Maulana R, Helms-Lorenz M. No aspect of structure should be left behind in relation to student autonomous motivation. Br J Educ Psychol. 2022;92(3):1086–108. doi: 10.1111/bjep.12489 35170032

[104] Dong W, Xiang C, Kamaruddin AY, Ali SKS, Yang Z, Wang X. The relationship between perceived teacher relatedness-support behavior (RSB) and learning motivation of dancesport students in universities. Sci Rep. 2024;14(1):28043. doi: 10.1038/s41598-024-79507-8 39543230 PMC11564526

[105] Slemp GR, Field JG, Ryan RM, Forner VW, Van den Broeck A, Lewis KJ. Interpersonal supports for basic psychological needs and their relations with motivation, well-being, and performance: A meta-analysis. J Pers Soc Psychol. 2024;127(5):1012–37. doi: 10.1037/pspi0000459 38635183

[106] Wang J, Liu R-D, Ding Y, Xu L, Liu Y, Zhen R. Teacher’s Autonomy Support and Engagement in Math: Multiple Mediating Roles of Self-efficacy, Intrinsic Value, and Boredom. Front Psychol. 2017;8:1006. doi: 10.3389/fpsyg.2017.01006 28690560 PMC5481391

[107] Zhou L, Gao Y, Hu J, Tu X, Zhang X. Effects of perceived teacher support on motivation and engagement amongst Chinese college students: Need satisfaction as the mediator. Front Psychol. 2022;13:949495. doi: 10.3389/fpsyg.2022.949495 36092093 PMC9455222

